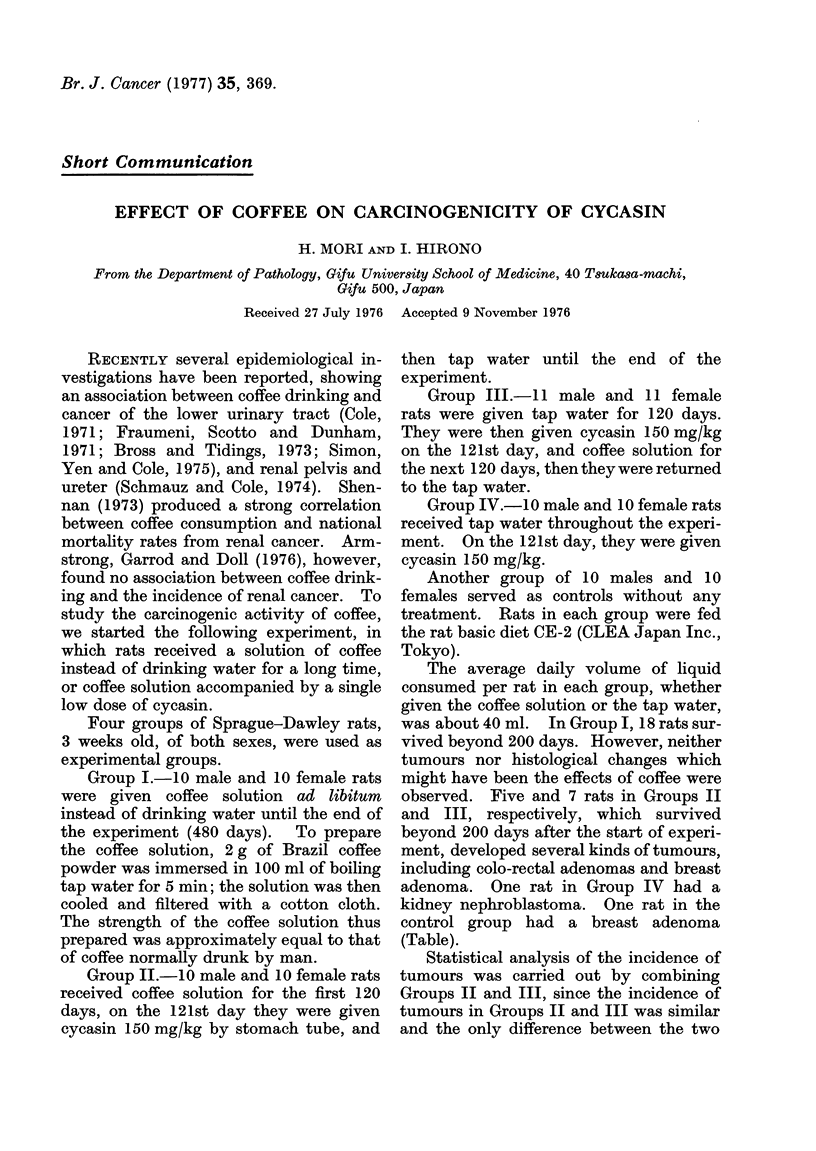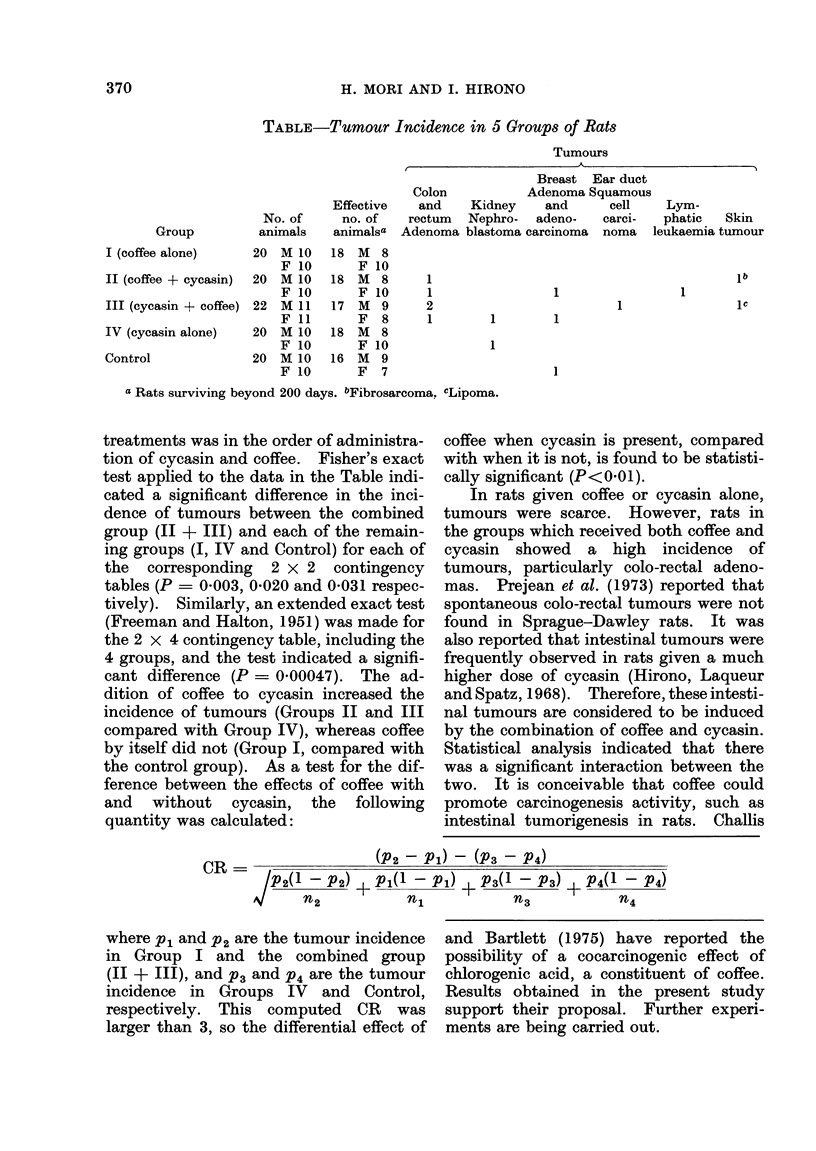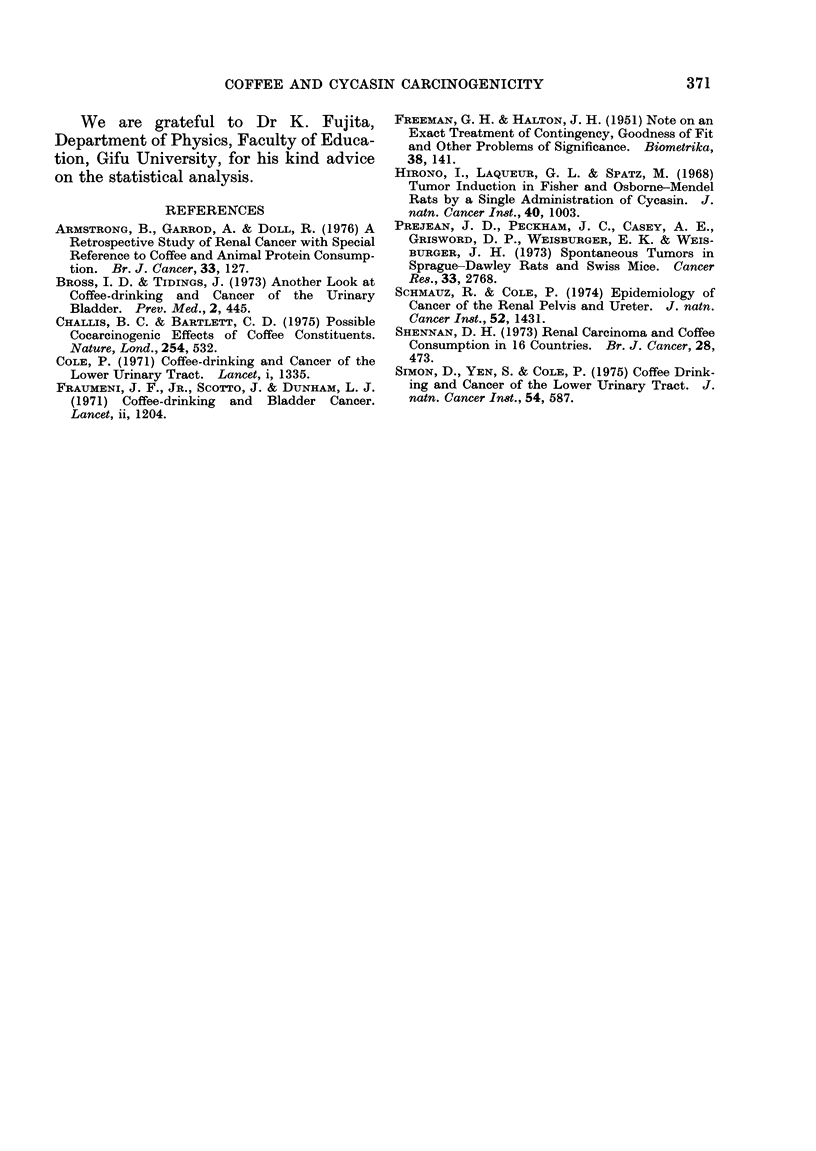# Effect of coffee on carcinogenicity of cycasin.

**DOI:** 10.1038/bjc.1977.51

**Published:** 1977-03

**Authors:** H. Mori, I. Hirono


*Br. J. Cancer* (1977) **35**, 369.

***Short Communication***

### EFFECT OF COFFEE ON CARCINOGENICITY OF CYCASIN

H. MORI AND I. HIRONO

*From the Department of Pathology, Gifu University School of Medicine,* 40 *Tsukasa-machi, Gifu* 500, *Japan*

Received 27 July 1976 Accepted 9 November 1976

RECENTLY several epidemiological investigations have been reported, showing an association between coffee drinking and cancer of the lower urinary tract (Cole, 1971; Fraumeni, Scotto and Dunham, 1971; Bross and Tidings, 1973; Simon, Yen and Cole, 1975), and renal pelvis and ureter (Schmauz and Cole, 1974). Shennan (1973) produced a strong correlation between coffee consumption and national mortality rates from renal cancer. Armstrong, Garrod and Doll (1976), however, found no association between coffee drinking and the incidence of renal cancer. To study the carcinogenic activity of coffee, we started the following experiment, in which rats received a solution of coffee instead of drinking water for a long time, or coffee solution accompanied by a single low dose of cycasin.

Four groups of Sprague–Dawley rats, 3 weeks old, of both sexes, were used as experimental groups.

Group I.—10 male and 10 female rats were given coffee solution *ad libitum* instead of drinking water until the end of the experiment (480 days). To prepare the coffee solution, 2 g of Brazil coffee powder was immersed in 100 ml of boiling tap water for 5 min; the solution was then cooled and filtered with a cotton cloth. The strength of the coffee solution thus prepared was approximately equal to that of coffee normally drunk by man.

Group II.—10 male and 10 female rats received coffee solution for the first 120 days, on the 121st day they were given cycasin 150 mg/kg by stomach tube, and then tap water until the end of the experiment.

Group III.—11 male and 11 female rats were given tap water for 120 days. They were then given cycasin 150 mg/kg on the 121st day, and coffee solution for the next 120 days, then they were returned to the tap water.

Group IV.—10 male and 10 female rats received tap water throughout the experiment. On the 121st day, they were given cycasin 150 mg/kg.

Another group of 10 males and 10 females served as controls without any treatment. Rats in each group were fed the rat basic diet CE-2 (CLEA Japan Inc., Tokyo).

The average daily volume of liquid consumed per rat in each group, whether given the coffee solution or the tap water, was about 40 ml. In Group I, 18 rats survived beyond 200 days. However, neither tumours nor histological changes which might have been the effects of coffee were observed. Five and 7 rats in Groups II and III, respectively, which survived beyond 200 days after the start of experiment, developed several kinds of tumours, including colo-rectal adenomas and breast adenoma. One rat in Group IV had a kidney nephroblastoma. One rat in the control group had a breast adenoma (Table).

Statistical analysis of the incidence of tumours was carried out by combining Groups II and III, since the incidence of tumours in Groups II and III was similar and the only difference between the two

TABLE—*Tumour Incidence in 5 Groups of Rats*

| Group | No. of animals | | Effective no. of animals[a] | | Tumours: Colon and rectum Adenoma | Kidney Nephro-blastoma | Breast Adenoma and adeno-carcinoma | Ear duct Squamous cell carci-noma | Lym-phatic leukaemia | Skin tumour |
|---|---|---|---|---|---|---|---|---|---|---|
| I (coffee alone) | 20 | M 10 | 18 | M 8 | | | | | | |
| | | F 10 | | F 10 | | | | | | |
| II (coffee + cycasin) | 20 | M 10 | 18 | M 8 | 1 | | | | | 1[b] |
| | | F 10 | | F 10 | 1 | | 1 | | 1 | |
| III (cycasin + coffee) | 22 | M 11 | 17 | M 9 | 2 | | | 1 | | 1[c] |
| | | F 11 | | F 8 | 1 | 1 | 1 | | | |
| IV (cycasin alone) | 20 | M 10 | 18 | M 8 | | | | | | |
| | | F 10 | | F 10 | | 1 | | | | |
| Control | 20 | M 10 | 16 | M 9 | | | | | | |
| | | F 10 | | F 7 | | | 1 | | | |

[a] Rats surviving beyond 200 days. [b]Fibrosarcoma. [c]Lipoma.

treatments was in the order of administration of cycasin and coffee. Fisher's exact test applied to the data in the Table indicated a significant difference in the incidence of tumours between the combined group (II + III) and each of the remaining groups (I, IV and Control) for each of the corresponding $2 \times 2$ contingency tables ($P = 0{\cdot}003$, $0{\cdot}020$ and $0{\cdot}031$ respectively). Similarly, an extended exact test (Freeman and Halton, 1951) was made for the $2 \times 4$ contingency table, including the 4 groups, and the test indicated a significant difference ($P = 0{\cdot}00047$). The addition of coffee to cycasin increased the incidence of tumours (Groups II and III compared with Group IV), whereas coffee by itself did not (Group I, compared with the control group). As a test for the difference between the effects of coffee with and without cycasin, the following quantity was calculated:

$$CR = \frac{(p_2 - p_1) - (p_3 - p_4)}{\sqrt{\frac{p_2(1-p_2)}{n_2} + \frac{p_1(1-p_1)}{n_1} + \frac{p_3(1-p_3)}{n_3} + \frac{p_4(1-p_4)}{n_4}}}$$

where $p_1$ and $p_2$ are the tumour incidence in Group I and the combined group (II + III), and $p_3$ and $p_4$ are the tumour incidence in Groups IV and Control, respectively. This computed CR was larger than 3, so the differential effect of coffee when cycasin is present, compared with when it is not, is found to be statistically significant ($P < 0{\cdot}01$).

In rats given coffee or cycasin alone, tumours were scarce. However, rats in the groups which received both coffee and cycasin showed a high incidence of tumours, particularly colo-rectal adenomas. Prejean *et al.* (1973) reported that spontaneous colo-rectal tumours were not found in Sprague–Dawley rats. It was also reported that intestinal tumours were frequently observed in rats given a much higher dose of cycasin (Hirono, Laqueur and Spatz, 1968). Therefore, these intestinal tumours are considered to be induced by the combination of coffee and cycasin. Statistical analysis indicated that there was a significant interaction between the two. It is conceivable that coffee could promote carcinogenesis activity, such as intestinal tumorigenesis in rats. Challis and Bartlett (1975) have reported the possibility of a cocarcinogenic effect of chlorogenic acid, a constituent of coffee. Results obtained in the present study support their proposal. Further experiments are being carried out.

We are grateful to Dr K. Fujita, Department of Physics, Faculty of Education, Gifu University, for his kind advice on the statistical analysis.

### REFERENCES

ARMSTRONG, B., GARROD, A. & DOLL, R. (1976) A Retrospective Study of Renal Cancer with Special Reference to Coffee and Animal Protein Consumption. *Br. J. Cancer*, **33**, 127.

BROSS, I. D. & TIDINGS, J. (1973) Another Look at Coffee-drinking and Cancer of the Urinary Bladder. *Prev. Med.*, **2**, 445.

CHALLIS, B. C. & BARTLETT, C. D. (1975) Possible Cocarcinogenic Effects of Coffee Constituents. *Nature, Lond.*, **254**, 532.

COLE, P. (1971) Coffee-drinking and Cancer of the Lower Urinary Tract. *Lancet*, i, 1335.

FRAUMENI, J. F., JR., SCOTTO, J. & DUNHAM, L. J. (1971) Coffee-drinking and Bladder Cancer. *Lancet*, ii, 1204.

FREEMAN, G. H. & HALTON, J. H. (1951) Note on an Exact Treatment of Contingency, Goodness of Fit and Other Problems of Significance. *Biometrika*, **38**, 141.

HIRONO, I., LAQUEUR, G. L. & SPATZ, M. (1968) Tumor Induction in Fisher and Osborne–Mendel Rats by a Single Administration of Cycasin. *J. natn. Cancer Inst.*, **40**, 1003.

PREJEAN, J. D., PECKHAM, J. C., CASEY, A. E., GRISWORD, D. P., WEISBURGER, E. K. & WEISBURGER, J. H. (1973) Spontaneous Tumors in Sprague–Dawley Rats and Swiss Mice. *Cancer Res.*, **33**, 2768.

SCHMAUZ, R. & COLE, P. (1974) Epidemiology of Cancer of the Renal Pelvis and Ureter. *J. natn. Cancer Inst.*, **52**, 1431.

SHENNAN, D. H. (1973) Renal Carcinoma and Coffee Consumption in 16 Countries. *Br. J. Cancer*, **28**, 473.

SIMON, D., YEN, S. & COLE, P. (1975) Coffee Drinking and Cancer of the Lower Urinary Tract. *J. natn. Cancer Inst.*, **54**, 587.